# A Robust and Efficient Production and Purification Procedure of Recombinant Alzheimers Disease Methionine-Modified Amyloid-β Peptides

**DOI:** 10.1371/journal.pone.0161209

**Published:** 2016-08-17

**Authors:** Marie Hoarau, Yannick Malbert, Romain Irague, Christelle Hureau, Peter Faller, Emmanuel Gras, Isabelle André, Magali Remaud-Siméon

**Affiliations:** 1 Laboratoire d’Ingénierie des Systèmes Biologiques et Procédés, Université de Toulouse, CNRS, INRA, INSA, Toulouse, France; 2 Laboratoire de Chimie de Coordination, CNRS, Université de Toulouse, INPT, Toulouse, France; National Center for Geriatrics and Gerontology, JAPAN

## Abstract

An improved production and purification method for Alzheimer’s disease related methionine-modified amyloid-β 1–40 and 1–42 peptides is proposed, taking advantage of the formation of inclusion body in *Escherichia coli*. A Thioflavin-S assay was set-up to evaluate inclusion body formation during growth and optimize culture conditions for amyloid-β peptides production. A simple and fast purification protocol including first the isolation of the inclusion bodies and second, two cycles of high pH denaturation/ neutralization combined with an ultrafiltration step on 30-kDa cut-off membrane was established. Special attention was paid to purity monitoring based on a rational combination of UV spectrophotometry and SDS-PAGE analyses at the various stages of the process. It revealed that this chromatography-free protocol affords good yield of high quality peptides in term of purity. The resulting peptides were fully characterized and are appropriate models for highly reproducible *in vitro* aggregation studies.

## Introduction

With a worldwide incidence of millions people, Alzheimer’s disease is considered as a major health issue, and much research effort is currently being devoted to better understand the causes and mechanisms of the disease. Diverse therapeutic approaches are being explored, including hormonotherapy [1], immunotherapy [2], genetics [3] or pharmaceutics [4].

Although their implication in the disease remains unclear, the presence of amyloid plaques and oligomers in the synaptic cleft is widely considered as one of the hallmarks of Alzheimer’s disease, and were found to be highly deleterious for neuron plasticity [5]. These aggregates are mainly constituted of amyloid-β (Aβ) peptides, namely Aβ_1–40_ and Aβ_1–42_, resulting from the abnormal proteolytic cleavage of the amyloid precursor protein. Understanding the mechanism of formation and the behavior of these aggregates could pave the way to new therapeutic approaches against Alzheimer’s disease, promoting multiple investigations on Aβ peptide aggregation process [6].

For *in vitro* experimentation, disposing of Aβ samples displaying reproducible behaviours is a key issue. Indeed, aggregation was shown to be highly sensitive to many factors, such as temperature, concentration, agitation, but also storage or sample preparation [7,8]. But most importantly, the presence of both contaminants and pre-aggregated forms of the peptide were shown to strongly alter the course of aggregation, either inhibiting it or yielding no fibrillary material. In this regard, the easy access to pure and homogeneous Aβ samples is still challenging and essential for *in vitro* experimentation.

Most investigations are usually performed with chemically synthesized peptides. Although convenient and easily accessible, solid phase peptide synthesis can be complex in terms of purification. Indeed, the presence of peptides with altered sequence caused by non-quantitative synthetic steps or of racemized peptides is observed, which can hardly be removed, even by up-to-date purification methods. Traces of salts or metal ions were also reported [9]. Finally, presence of pre-formed aggregates due to storage conditions is frequent. All of this induce significant variations of aggregation properties from batch to batch. This has been recently exemplified in a study, which showed that the recombinant peptide is more aggregation-prone and more neurotoxic than its synthetic analogue, demonstrating a deleterious effect of synthetic procedures on peptide properties [10]. This has encouraged the exploration of various biological routes to access recombinant Aβ, getting rid of such issues.

Aβ peptide recombinant production was attempted in yeast [11] or by combining recombinant and synthetic procedures [12] but, to date, the most common expression system remains *Escherichia coli*. Most often, peptides are appended to a fusion protein that enhance their solubility and ease their isolation and purification using affinity chromatography. However, such strategies raised several issues. First of all, despite the increased solubility of the fusion proteins, a significant amount of the peptide frequently remains insoluble, thus requiring an extra denaturation step to achieve full recovery [13,14]. Moreover, a cleavage site has to be installed, its position being critical; indeed if not carefully chosen, upon cleavage amino-acids will be introduced at the terminal end of the peptide, potentially modifying its aggregation and metal-binding properties [15,16]. The same drawback is also observed with smaller affinity tags [17,18]. This problem has been solved through the use of proteolytic enzymes whose cleavage sites enable the formation of native Aβ (Factor Xa, Enterokinase…) [19,20]. Still such methods are time-consuming and require many purification steps, including long affinity chromatography purification procedures that induce in a loss of peptide. A representative example was described by Zhang and co-workers, showing that only 24% of Glutathione S-transferase-tagged peptide is recovered in the soluble fraction after cell lysis. This yield is weakly affected by affinity chromatography (21%), but drops to 5.1% after thrombin cleavage. To overcome this drawback, it was proposed to express Aβ in its native form in *E*. *coli* [21,22]. Notably, Aβ forms insoluble inclusion bodies (IBs) when expressed in *E*. *coli* in their native form. This was exploited by Walsh and co-workers to isolate pure recombinant methionine-modified Aβ peptides MAβ_1–40_ and MAβ_1–42_ in five purification steps [21].

We describe here an alternative and straightforward procedure of recombinant MAβ_1–40_ and MAβ_1–42_ production and purification. First, expression conditions were optimized using Thioflavin-S (Th-S), a known fluorescent probe for *in vivo* imaging of IBs. We subsequently established a rapid purification process based on inclusion body treatment with NaOH followed by an ultrafiltration step that has proven remarkably efficient to remove protein and nucleic acid contaminants. Finally, a detailed monitoring of the purification process was conducted, showing that only a combination of characterization techniques can ensure sample reliability.

## Materials and Methods

All chemicals were purchased from Sigma-Aldrich (St. Louis, USA). pET28a plasmid was purchased from Novagen (Darmstadt, Germany). NcoI, XhoI and BamHI restriction enzymes Antarctic phosphatase and T4 DNA Ligase were purchased from New England Biolabs (Ipswich, USA) and used with the provided buffer according to supplier’s recommendations. pET28a_His-Thromb-Aβ_1–42_ plasmid was synthesized by Genecust (Dudelange, Luxembourg). *E*. *coli* TOP10 cells and *E*. *coli* BL21 (DE3) cells were obtained from Invitrogen (Carlsbad, USA). QIAprep spin MiniPrep kit was purchased from Qiagen (Hilden, Germany). Sequence analyses of the constructions were performed by GATC Biotech (Constance, Germany).

### Vectors construction and cloning procedure

The commercial pET28a plasmid was digested by NcoI and XhoI restriction enzymes, dephosphorylated using Antarctic phosphatase, and gel purified on an agarose 0.8% Tris acetate EDTA (TAE) gel. The pET28a_His-Thromb-Aβ_1–42_ plasmid was used as template for PCR amplification of the Aβ_1–42_ coding sequence using pR_pET28_Ab42: AGCGGTGGCAGCAGCCAACTCAGCT and pF_pET28_Ab42: GACCTACCCATGGACGCTGAATTTCGCCACGACTCCGGCTAT as primers. The PCR product was digested by NcoI and XhoI, gel purified on an agarose 1.2% TAE gel and ligated in the NcoI/XhoI digested pET28a yielding the desired pET28a_MAβ_1–42_ plasmid. The construction was used to transform chimiocompetent *E*.*coli* TOP10 cells that were grown on solid Luria-Bertani (LB) medium (10 g/L tryptone, 10 g/L NaCl, 5 g/L yeast extract) containing 50 mg/L kanamycin (1X). One colony was used to inoculate 5 mL LB kanamycin 1X at 37°C. Cells were grown overnight, harvested before plasmid extraction using a MiniPrep kit.

The pET28a_MAβ_1–40_ was obtained from pET28a_MAβ_1–42_ using a similar procedure except that the sequence coding for Aβ_1–40_ was PCR-amplified with pR_pet28_Ab40: AATGGATCCTAATTAAACAACGCCGCCAACCATCAGACCGATG and pF_pet28_Ab40: AATTGTGAGCGGATAACAATTCCCCTCTAG as primers. The PCR-product was digested using NcoI and BamHI, and ligated into NcoI/BamHI digested and dephosphorylated pET28a.

### Expression of MAβ_1–40_ and MAβ_1–42_ and inclusion body isolation

Chemically competent *E*. *coli* BL21 (DE3) cells transformed with pET28a_MAβ_1–40_ or pET28a_MAβ_1–42_ plasmids were used for peptide production. One single colony was used to inoculate 10 mL LB kanamycin 1X medium. The cells were grown for 16 h. 100 mL of LB medium containing kanamycin 1X were inoculated to an OD_600_ of 0.05 with an overnight pre-culture performed from one single clone in 10 mL LB-Kana medium. Cells were grown at 37°C under agitation to an OD_600_ of 0.8. Expression was induced with 0.5 mM Isopropyl β-*D*-1-thiogalactopyranoside (IPTG) during for 4 additional hours of culture at 37°C. Cells were harvested by centrifugation (8,000 g, 10 min) and re-suspended to an OD_600_ of 80 in lysis buffer (50 mM Tris-HCl, 100 mM NaCl, pH 8) containing 1% Tergitol-type NP40 detergent. Cells were then lysed by 30s sonication on ice and the lysate was centrifuged (12,000 g, 10 min). The insoluble fraction was re-suspended in 1 mL lysis buffer, containing phenylmethanesulfonyl fluoride (15 mM) and lysozyme (300 μg/mL), and incubated for 1 h at room temperature. IBs were harvested by centrifugation (12,000 g, 10 min), washed with Triton X100 0.5% and twice with phosphate saline buffer (PBS) 1X pH 8. IBs were finally resuspended in PBS buffer and submitted to 10 cycles of 10 s sonication followed by centrifugation to remove nucleic acid contaminants contained in the supernatant and that could be stacked on their surface, according to a previously reported procedure [23].

### Peptide purification

IBs were denatured by 2 h incubation in 500 μL of NaOH 50 mM under smooth shaking at room temperature. The resulting mixture was centrifuged (12,000 g, 5 min), and the supernatant neutralized by addition of 500 mM HCl. The formation of a white precipitate was observed. The mixture was centrifuged once again, and the supernatant was collected. To optimize recovery, this denaturation/neutralisation cycle was applied twice. Supernatants were passed through a 30-kDa Amicon-Ultra centrifugal device, to remove high molecular weight contaminant proteins, and then concentrated on a 3-kDa Amicon-Ultra centrifugal device.

### SDS-PAGE analysis

15 μL protein samples were mixed with 5 μL Laemli Sample buffer (Bio-Rad) containing 10% β-mercaptoethanol, and denatured at 95°C for 10 min. Samples were then loaded on a Mini-protean TGX Stain-Free Any Kd Precast Gel (Bio-Rad) and electrophoresis was run for 30 min at 150 V in Tris/Glycine/SDS buffer (Bio-Rad). Gel was then stained using PageBlue Protein Staining solution (Bio-Rad).

### Thioflavin-S staining

Samples of Aβ peptide producing cells or purified IBs are centrifuged (12,000 g, 10 min) and resuspended in 200 μL Thioflavin-S 125 μM in PBS 1X pH 8. After 15 min incubation, cells or IBs were harvested by centrifugation, washed with 200 μL PBS, re-suspended in PBS, and submitted to fluorescence experiments.

### Fluorescence microscopy

50 μL samples of Th-S stained cells or IBs were dispensed on a microscope glass slide and imaged using a Leica microscope with a 63x lens with a DAPI filter (λ_exc_ = 340–380 nm, dichroic = 400, λ_em_ = 425 nm).

### Fluorescence spectrophotometry

1 mL samples of Th-S stained cells or IBs were placed in a quartz cuvette and their emission spectra were recorded using a Jasco fluorimeter (λ_exc_ = 375 nm). Right after each measurement, OD_600_ was measured, to normalize the signal.

### ^1^H NMR

Pure samples of peptides in water were diluted to 100 μM in phosphate buffer 50 mM pH 7 in D_2_O. ^1^H NMR spectra were recorded on a 500 MHz NMR (Bruker) at 25°C using solvent as reference. As a control, synthetic Aβ_1–40_ was dissolved in NaOD and diluted to 100 μM under the same conditions.

### LC-MS

Peptide samples were analysed by LC-MS on an Agilent 1200 HPLC fitted with a Macherey Nagel Nucleodur 300–5 C4 ec 250 mm x 2 mm 5 μm at 30°C coupled with a Thermo Fisher Scientific LCQ Fleet mass spectrometer fitted with an H-ESI II Probe.

Samples were eluted at 0.33 mL/min using a gradient of acetonitrile going from 5% to 90% in aqueous formic acid 0.1% in 12 min. Multiply-charged peptides were detected by mass spectrometry (ESI +).

### Thioflavin-T aggregation assay

To ensure of the homogeneity of the starting sample, monomeric peptides were isolated by steric exclusion chromatography (SEC). An Aktä Purifier system equipped with a Superdex 75 10/300 column was equilibrated with 2 column volumes of 15 mM NaOH. Peptide samples in NaOH 50 mM were then eluted using NaOH 15 mM at 1 mL/min, with UV monitoring at 220 and 293 nm. Monomeric MAβ peptides display retention times around 9 min. Fractions containing peptides were collected, centrifuged, and their concentration was assessed by UV-Vis at pH 12 (λ = 293 nm, ε = 2400 L.mol^-1^.cm^-1^).

In a 384-wells microplate (Greiner) were added phosphate buffer (50 mM), Thioflavin-T (10 μM) and freshly SEC-purified peptide (20 μM or 50 μM). Fluorescence intensity was recorded every 5 min for a total duration of 4 days using a ClarioStar plate reader (BMG Labtech) set at 37°C. Fluorescence parameters were set as follows: λ_exc_ = 440 nm, λ_em_ = 490 nm, gain = 650, shaking at 200 rpm for 12 s before each measurement. Aggregation curves were fitted using KaleidaGraph software.

## Results and Discussion

### Optimization of MAβ_1–40_ and MAβ_1–42_ expression

The genes encoding MAβ_1–40_ and MAβ_1–42_ were first cloned in pET28. They were then expressed by growing *E*. *coli* BL21 (DE3) transformed with the recombinant plasmids on LB broth supplemented with kanamycin. To determine optimal conditions for peptide expression, we envisioned a procedure based on Th-S monitoring.

**Fig 1:**
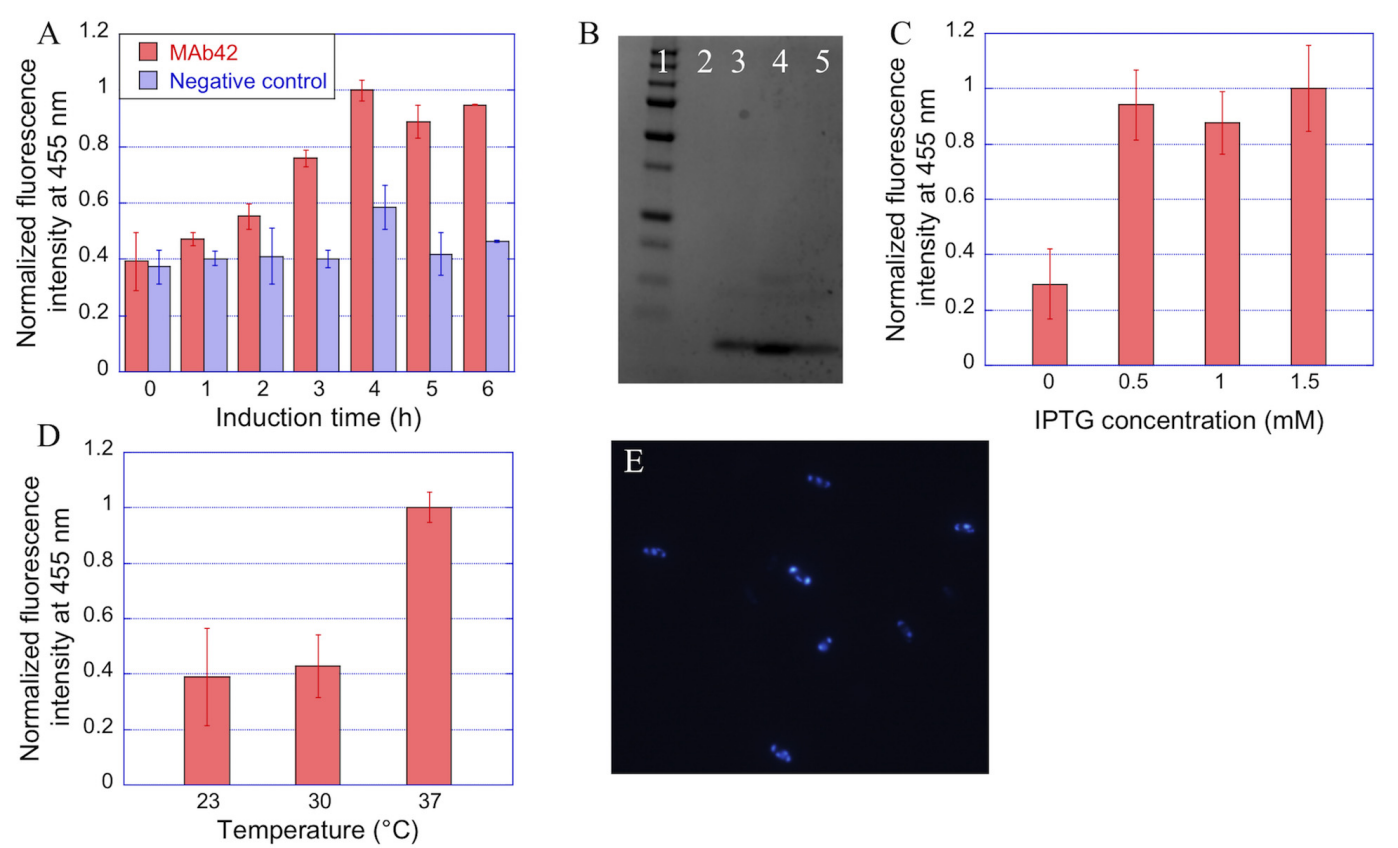
MAβ_1–42_ expression monitoring using Thioflavin-S. **A**. Variations of normalized fluorescence of Th-S-treated *E*. *coli* cells expressing MAβ_1–42_ with culture induction time. **B**. SDS-PAGE of inclusion bodies formed during *E*. *coli* cultures expressing MAβ_1–42_; lane 1: 10–250 kDa protein ladder, lane 2 to 5: samples taken at 0, 2, 4, and 6 hours of growth, respectively. **C.** Normalized fluorescence intensity of Th-S-treated *E*. *coli* cells expressing MAβ_1–42_ as a function of IPTG concentration used for induction. **D**. Normalized fluorescence intensity of Th-S-treated *E*. *coli* cells expressing MAβ_1–42_ during 4h at various temperatures. **E**. Fluorescence microscopy of *E*. *coli* cells expressing MAβ_1–42_ as IBs after 4 h culture.

Indeed, Th-S is usually used for staining amyloid plaques but was also recently shown to stain bacterial IBs formed by MAβ peptides, both *in vivo* and *in vitro*. Hence, Th-S was used to detect protein aggregation in bacteria [22], or to screen *in vivo* aggregation inhibitors [24]. In order to use Th-S staining as a tool for the optimization of peptide expression, the correlation between IB content and the Th-S fluorescence level had to be established. For this purpose, cultures expressing MAβ_1–42_ were stopped at different induction times. The cells were sampled, incubated with Th-S, and their emission spectra were recorded. As shown in Fig 1A, the fluorescence level increased during the first 4 hours of induction and then reached a plateau. The corresponding cultures were lysed and peptide was purified and analysed by SDS-PAGE. A good correlation was observed between the amount of purified peptide and the fluorescence level (Fig 1B). Similar results were obtained with MAβ_1–40_, showing that Th-S staining is very useful to estimate MAβ IB content in cells. Based on these results, a 4 h induction was considered as adapted and applied throughout the rest of the study.

Th-S monitoring was then used to screen for the optimal concentration of IPTG inducer. No significant variations of IB production was observed with IPTG concentration comprised between 0.5 and 1.5 mM (Fig 1C). Contrariwise, increased levels of protein contaminants were observed when using 1 or 1.5 mM IPTG, which could be due to the overexpression of *E*. *coli* chaperones [25]. Induction at 0.5 mM IPTG was thus found optimal. Finally, the optimal temperature of cell growth was investigated. As shown in Fig 1D, the best temperature allowing the formation of a maximal amount of IBs was 37°C.

This preliminary work allowed us to establish the optimal conditions of MAβ peptide recombinant production as corresponding to recombinant cell cultures carried out at 37°C and induced for 4 h with 0.5 mM IPTG. Under these conditions, both visible and fluorescence microscopy confirmed the presence of IBs located at the poles of the cells (Fig 1E).

### Purification of MAβ_1–40_ and MAβ_1–42_

As Aβ peptides were produced as IBs, the first step of the purification procedure consisted in isolating and purifying IBs. The main challenge of this approach was to succeed in removing proteins and nucleic acids that could be trapped in the IBs or stacked on their surface.

First, the previously reported “soni-removal” method, which consists in multiple sonication and centrifugation cycles, was performed to roughly eliminate nucleic acids and proteins stacked at the IB surface [23]. At this stage, Th-S fluorescence visualization of isolated IBs confirmed that their average size remained unchanged, showing no deterioration due to sonication.

**Fig 2:**
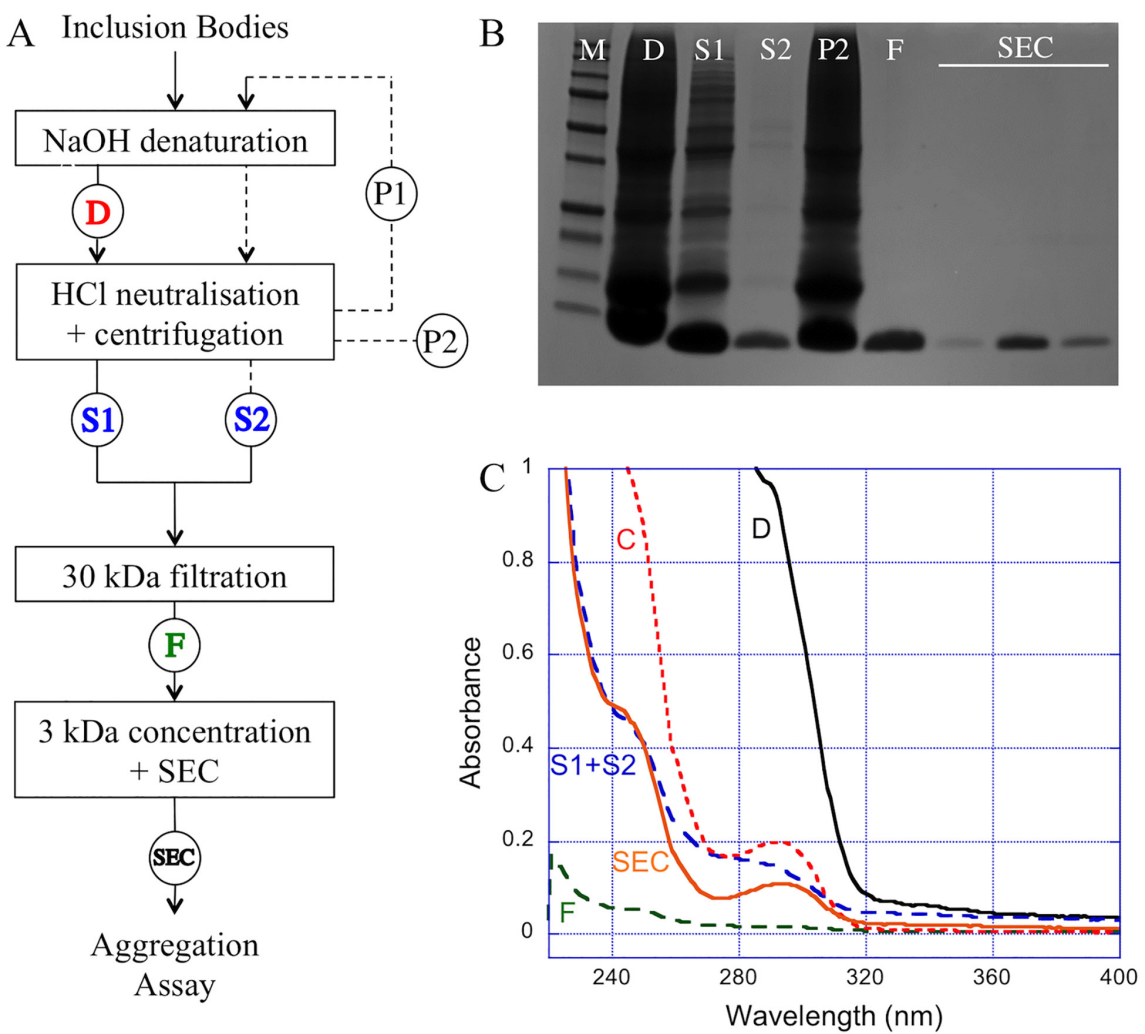
Purification monitoring of MAβ. **A.** Simplified scheme of the purification procedure. Circled letters correspond to samples collected for either UV/Vis measurements or gel analysis. Supernatant S1 and S2, and pellet P1 and P2 correspond to the supernatant and the precipitate obtained for the two cycles of denaturation/neutralisation. **B.** SDS-PAGE at different steps of purification, compared with 10–250 kDa protein marker (lane M). **C.** UV-Vis spectra at pH 12 after IBs denaturation (D), after denaturation/neutralisation cycles (S1+S2), after 30-kDa ultrafiltration (F), after concentration (C), and after size exclusion chromatography (SEC).

The protocol shown in Fig 2A was then applied. Aβ peptides are soluble at high pH, since their isoelectric point is about 5–6. The IBs were thus first incubated with 50 mM sodium hydroxide, to solubilize MAβ. Upon pH neutralization, a large amount of proteins is removed by precipitation, while MAβ peptides remain in the supernatant (Fig 2B, lanes S1 and P2). The high pH denaturation/neutralization cycle was applied twice to maximize MAβ recovery (Fig 2B, lane S2). Further completion of contaminant elimination was carried out by ultrafiltration of the solubilized peptides followed by concentration, respectively on a 30-kDa and a 3-kDa molecular weight cut-off membranes. As shown on the electrophoresis gel (Fig 2B, lane F), the ultrafiltration step allowed getting one single band around 5 kDa, the presence of any protein contaminant being excluded.

**Fig 3:**
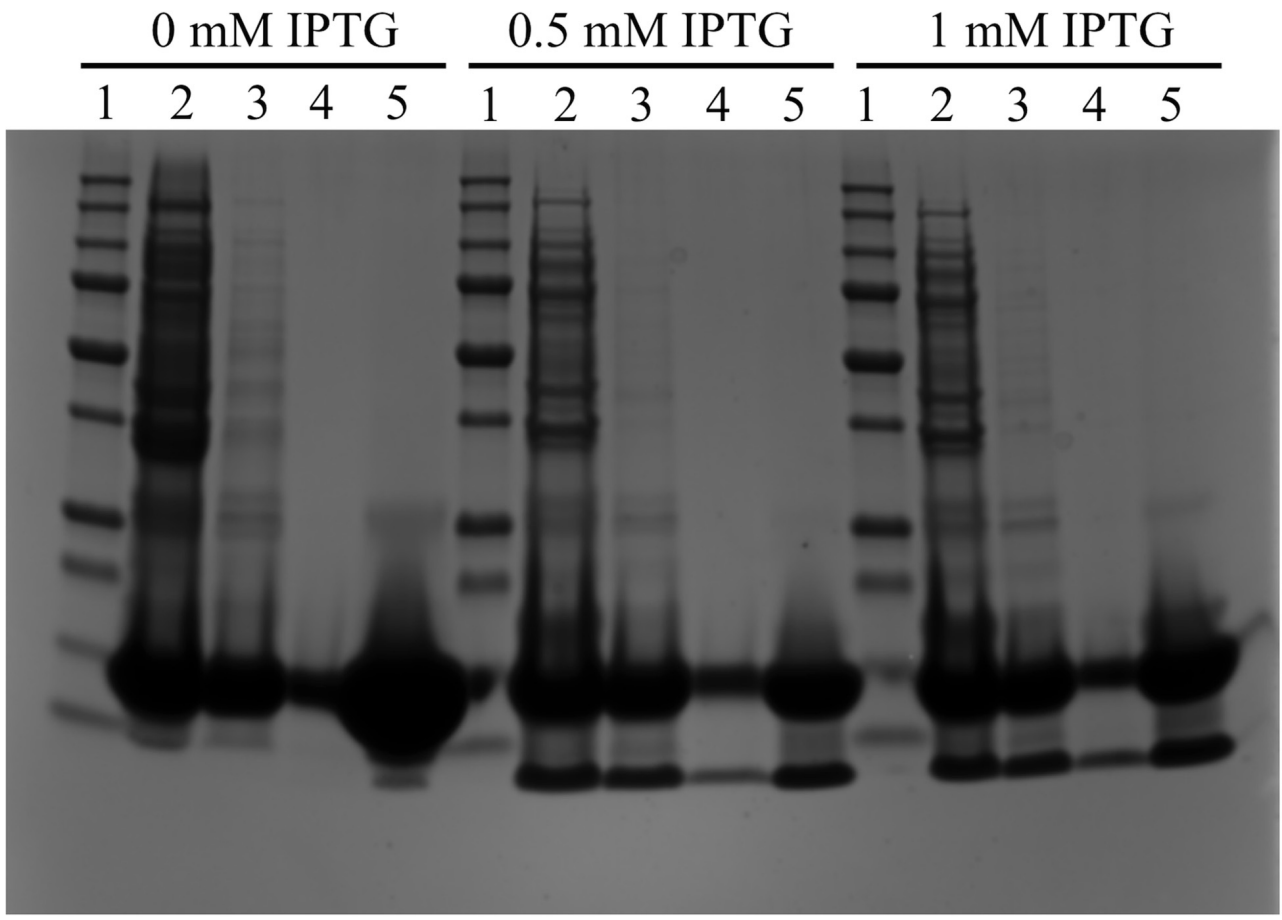
Purification protocol of MAβ_1–42_ IBs dissolved in 8M urea. Lane 1: 10–250 kDa protein marker, lane 2: IBs after urea denaturation, lane 3: after passing through DEAE resin, lane 4: after 30-kDa ultrafiltration, lane 5: after 3-kDa concentration.

In parallel, the MAβ_1–42_ IBs were also solubilized using 8M urea and then passed through a DEAE Sepharose ion-exchange resin to remove NA traces. As seen in Fig 3, this protocol did not allow the elimination of a 13 kDa protein contaminant, regardless of IPTG concentration used for induction. Furthermore, this protein could not be eliminated by ultrafiltration indicating that the urea treatment did not allow breaking the interaction probably existing between the 13 kDa protein and the peptide (Fig 3 lanes 4 and 5). This clearly showed the limitation of the denaturation with urea and emphasizes the interest of the NaOH treatment with to eliminate proteins and nucleic acids.

At this point, one important issue was the monitoring of the sample purity at the various stages of the purification procedure. The Bradford protein assay was eliminated because it does not give any information on sample purity and only allows the determination of a global protein content. Combination of UV-Vis spectrophotometry with gel electrophoresis was clearly preferred. Indeed, SDS-PAGE permits to visualize the presence of protein contaminants while UV-Vis analyses inform on the predominant presence of either nucleic acids (λ = 260 nm) or specific amino acids, namely tryptophan (λ = 280 nm), tyrosine/tyrosinate (λ = 274 nm/λ = 293 nm), phenylalanine (λ = 257 nm) [26]. Both methods were thus combined to assess the purity level of the peptide samples depending on the purification steps as shown in Fig 2C. After denaturation (Fig 2C, spectrum D), a lot of species are evidenced. The supernatant harvested after neutralization (Fig 2C, spectrum S1+S2), still contain proteins but is already devoid of nucleic acids. After ultrafiltration, the sample was too diluted to give an informative UV spectrum (Fig 2C, spectrum F). Finally, the concentration step yielded a pure sample showing a UV signature with the typical UV band at λ = 293 nm corresponding to the tyrosinate form (Fig 2C, spectrum C). This typical profile is conserved after size exclusion chromatography, which was performed to isolate only monomeric peptide before aggregation assays (Fig 2C, spectrum SEC). Overall, these analyses confirmed the high level of purity of the peptide preparation.

As the sample was devoid of any protein contaminants, the pH-dependent absorption of Tyr was used to estimate the peptide yield. Absorption spectra of the peptides were thus recorded at pH 12 (showing the absorption band of tyrosinate at 293 nm) and at pH 2 (displaying no tyrosinate band). The precise quantification of the tyrosine content of the sample was performed by subtracting spectra. This enabled the estimation of the peptide yield as being around 4 mg/L of culture, which is in the same range as those previously reported, comprised between 3 and 7 mg/L [13,14,19,27,28].

### Characterization of MAβ_1–40_ and MAβ_1–42_

**Fig 4:**
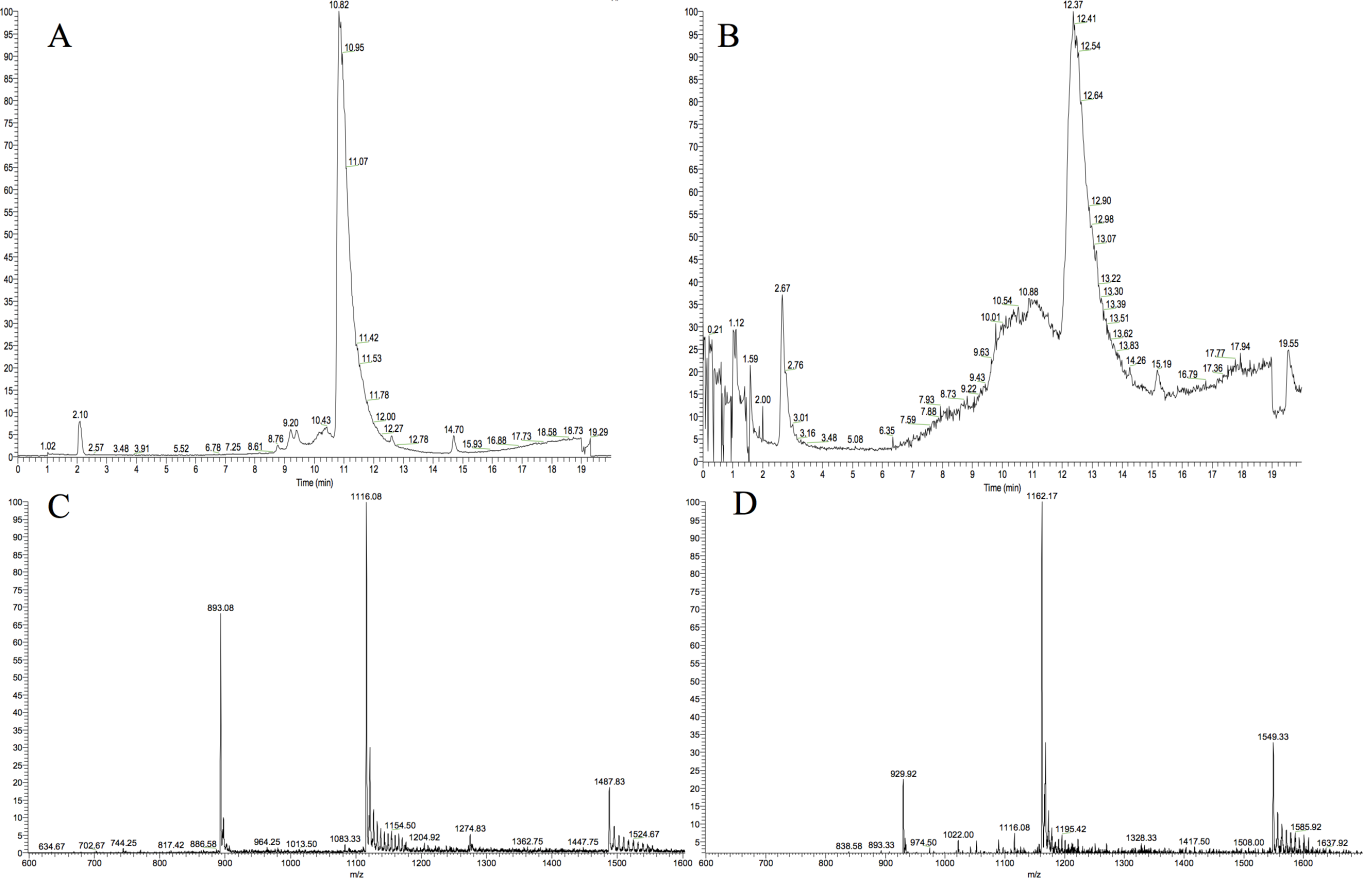
LC-MS analysis of MAβ_1–40_ and MAβ_1–42_. **A.** LC trace of MAβ_1–40_ monitored by mass detection. **B.** LC trace of MAβ_1–42_ monitored by mass detection. **C.** MS of MAβ_1–40_ displaying [M+5H]^5+^, [M+4H]^4+^, and [M+3H]^3+^ peaks (expected values: 892.65, 1115.56, 1487.07). **D.** MS of MAβ_1–42_ displaying [M+5H]^5+^, [M+4H]^4+^, and [M+3H]^3+^ peaks (expected values: 929.47, 1162.17, 1548.44).

To further characterize the peptides, LC-MS and NMR analyses were conducted right after the concentration step on both peptides. MAβ_1–40_ displays a clean chromatographic trace with a major peak at 10.8 min. The mass spectrum associated to this peak is in good agreement with the expected masses (Fig 4A and 4C). MAβ_1–42_ chromatogram is less resolved, the main peak being shouldered (Fig 4B and 4D). The masses detected for both the shoulder and the peak are consistent with the calculated masses of MAβ_1–42_, indicating that oligomeric forms of MAβ_1–42_ could be present. This is consistent with the high propensity of Aβ_1–42_ to aggregate.

**Fig 5:**
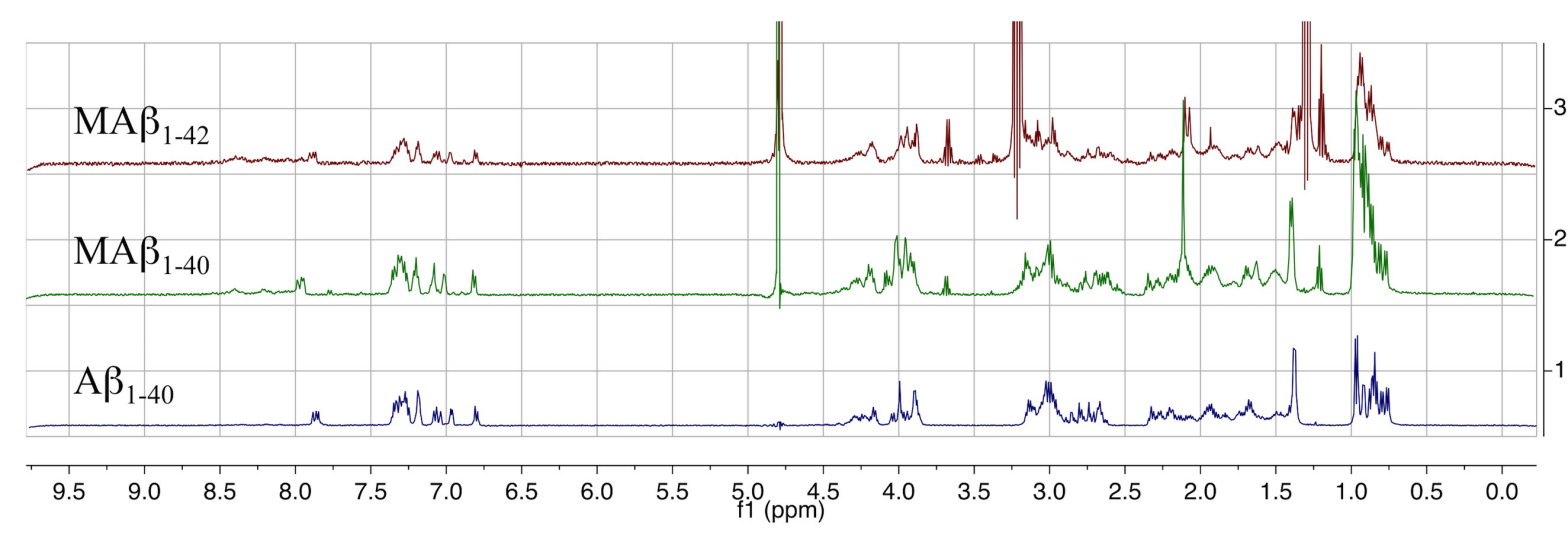
^1^H NMR of MAβ_1–40_ and MAβ_1–42_ compared with Aβ_1–40_. Samples of MAβ in water are diluted to 100 μM in 100 mM phosphate buffer pH 7 in D_2_O. Synthetic Aβ_1–40_ is dissolved in NaOD and diluted to 100 μM under the same conditions. Signals at 4.75 ppm correspond to H_2_O, and signals at 3.25 ppm and 1.25 ppm correspond to traces of ethanol.

^1^H NMR spectra of both peptides were then recorded, and compared with commercially available synthetic Aβ_1–40_ (Fig 5). They display the typical signals usually observed for Aβ_1–40_ peptide. In the aromatic region, signals corresponding to the Hδ and Hε of aromatic residues are well defined. As such, Phe 19 and 20 (*ca*. 7.2 ppm and *ca*. 7.3 ppm) and Tyr 10 (*ca*. 7.95 ppm and *ca*. 6.8 ppm) can be distinguished. Slight shifts are observed for His 6, 13 and 14 (*ca*. 7.8 ppm and *ca*. 7.1 ppm), which can be attributed to pH variations. The absence of signal *ca*. 7.5 ppm confirms the absence of Trp residue in the peptides. Expectedly, signals corresponding to Hα (4.5–3.8 ppm) and Hβ (3.2–2.6 ppm and 2.3–1.5 ppm) are more difficult to attribute in details. In the aliphatic region, signals corresponding to Lys 16 and 28 (1.4 ppm) and to Val and Leu (1.0–0.7 ppm) are observed. Finally, the signal at 3.9 ppm reveals the presence of a methionine residue for the recombinant peptides as expected.

**Fig 6:**
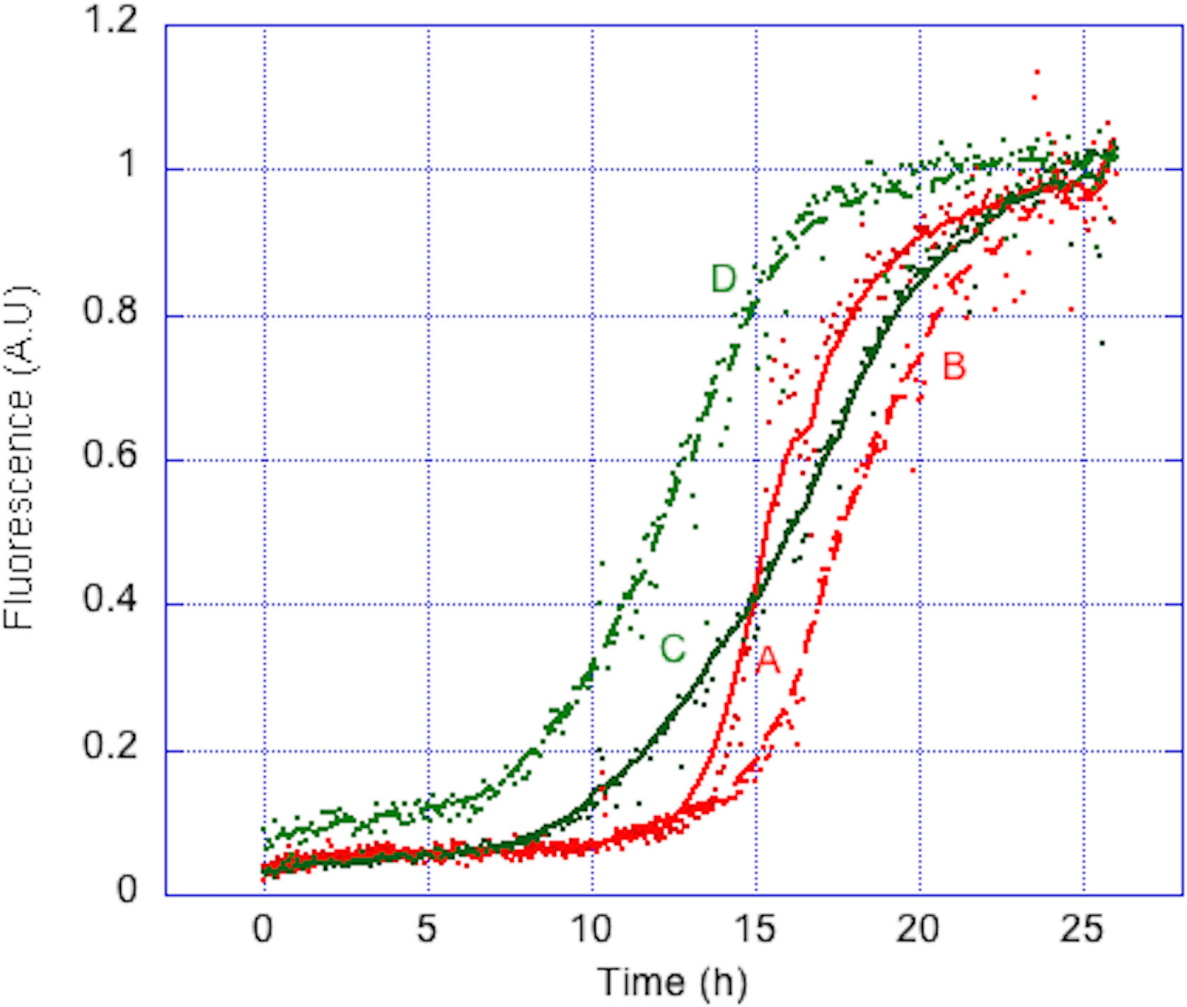
Normalized aggregation curves of MAβ_1–40_ and MAβ_1–42_. Two different batches of MAβ_1–40_ (A and B) and MAβ_1–42_ (C and D) (20 μM) aggregate in the presence of Th-T (10 μM) in phosphate buffer (50 mM, pH 7). Assay was run at 37°C with gentle shaking. Each experiment was run in triplicates.

Because most *in vitro* studies on Aβ peptides consist in studying their aggregative properties under various conditions, it was essential to ensure that the auto-assembling properties of the peptides are conserved after this purification process. Prior to this, it was important to remove traces of oligomeric species, to ensure a homogeneous aggregation process. The two peptides were thus submitted to SEC. To assess the apparent molecular weight of targeted peptides, a calibration curve was established using other unstructured peptides. It confirmed that the apparent molecular weight of the two peptides was about 4 kDa (S1 Fig). Fractions corresponding to monomeric peptides were then submitted to Th-T fibrillization assay. In 26 h, both peptides afford a characteristic sigmoidal aggregation curve, with aggregation half-times around 15 h (Fig 6). The MAβ peptides obtained through our purification procedure thus conserved their auto-assembling properties. The same results were obtained with peptide batches from different cultures, showing the high robustness of the purification procedure in terms of reproducibility. This purification protocol could thus be applied for routine lab-scale MAβ production. This also confirms that the presence of a *N*-ter Met residue does not affect the aggregation properties of MAβ peptides as previously reported [16]. Removing Met residue could be beneficial for specific studies such as metal coordination, but does not seem mandatory for classical aggregation assays.

## Conclusion

We have here reported a rapid, straightforward and highly reproducible recombinant production and purification of MAβ_1–40_ and MAβ_1–42_. First, we showed that Th-S staining was reliable to monitor IB production during cell growth. Purification monitoring provided evidence that only the combination of UV-Vis spectrophotometry and gel electrophoresis analyses could properly allow the assessment of the peptide purity along the purification process.

In addition, we established that pH treatment of IBs is an efficient way to get rid of most nucleic acid and protein contaminants, contrasting with commonly used chaotropic agents. Additional purification steps consisting in simple ultrafiltrations, providing clean peptide preparations, as revealed by UV-Vis spectrophotometry, SDS-PAGE, LC-MS and ^1^H NMR. With this protocol, up to 4 mg of peptides are produced within 2 days. In addition, the preparations are suitable for aggregation assays, which were highly reproducible from batch to batch, accounting for a robust purification procedure. Supplementary investigation might now be engaged to remove the *N*-ter methionine residue, in order to obtain the native Aβ, as already proposed by Walsh and co-workers and further improve the peptide yield [21].

## Supporting Information

S1 Fig SEC calibration curve with unstructured peptides. The different peptides were dissolved in NaOH 50 mM to 10 mg/mL and injected on a Superdex 75 10/300 GL. Samples were eluted with NaOH 15 mM at 1 mL/min monitoring at 220 nm and 293 nm. The arrows indicate the elution volume found for synthetic Aβ_1–40_ and the two recombinant MAβ_1–40_ and MAβ_1–42_ peptides.
(TIFF)
