# Correction: A Robust and Efficient Production and Purification Procedure of Recombinant Alzheimers Disease Methionine-Modified Amyloid-β Peptides

**DOI:** 10.1371/journal.pone.0205048

**Published:** 2018-09-26

**Authors:** Marie Hoarau, Yannick Malbert, Romain Irague, Christelle Hureau, Peter Faller, Emmanuel Gras, Isabelle André, Magali Remaud-Siméon

There are errors in the placement of references 17 and 18. The sixth sentence of the fifth paragraph in the Introduction section should have no citations. The seventh sentence should have cited references 17 and 18, in addition to references 19 and 20.

The correct sentences should read: The same drawback is also observed with smaller affinity tags. This problem has been solved through the use of proteolytic enzymes whose cleavage sites enable the formation of native Aβ (Factor Xa, Enterokinase …) [17], [18], [19], [20].
