# Supplementary figures and images for: A Robust and Efficient Production and Purification Procedure of Recombinant Alzheimers Disease Methionine-Modified Amyloid-β Peptides
Source: PLoS One. 2016 Aug 17;11(8):e0161209. doi: 10.1371/journal.pone.0161209 (PMC4988814; doi:10.1371/journal.pone.0161209)

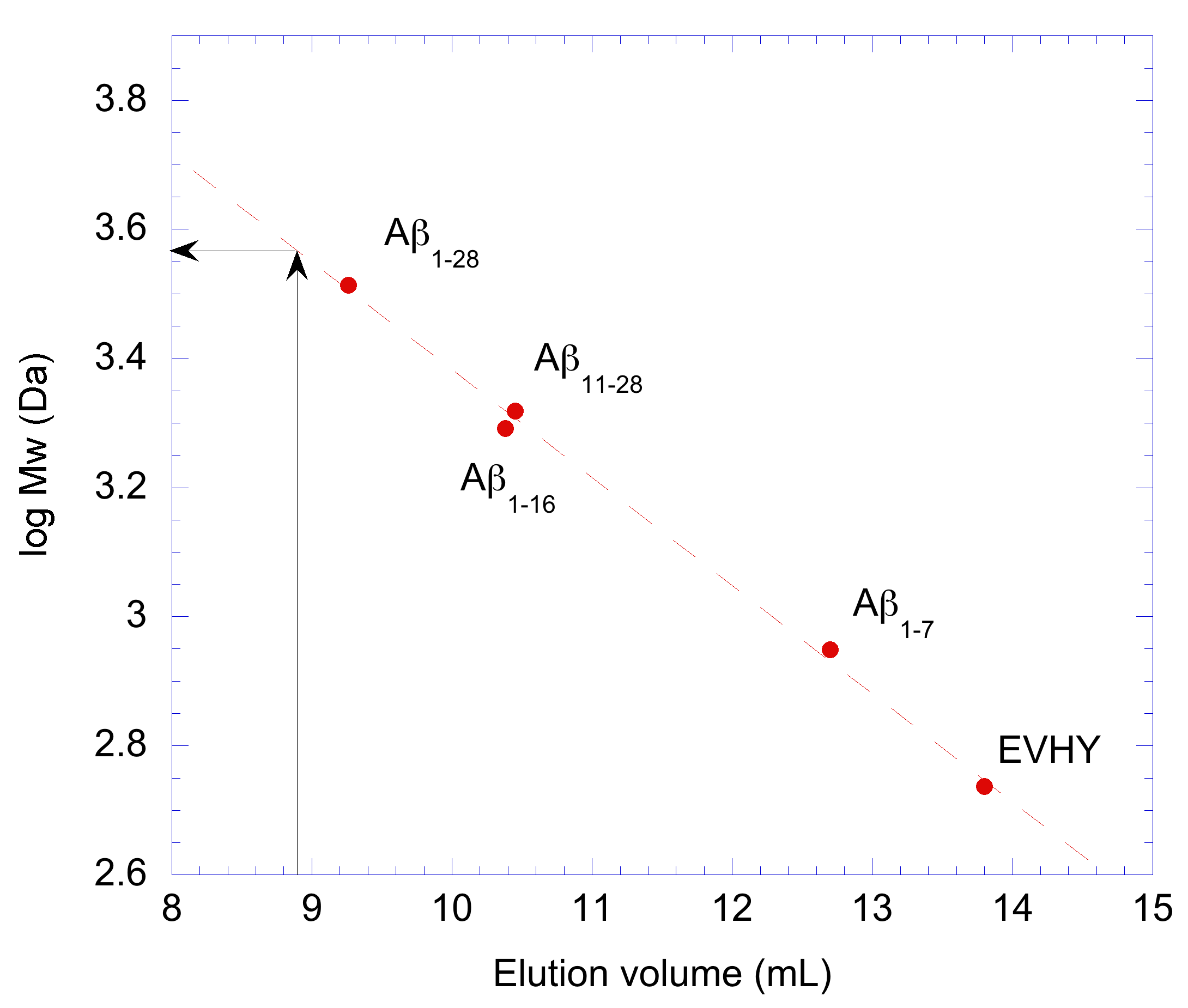

Supplement: S1 Fig — The different peptides were dissolved in NaOH 50 mM to 10 mg/mL and injected on a Superdex 75 10/300 GL. Samples were eluted with NaOH 15 mM at 1 mL/min monitoring at 220 nm and 293 nm. The arrows indicate the elution volume found for synthetic Aβ1–40 and the two recombinant MAβ1–40 and MAβ1–42 peptides. (TIFF) [file pone.0161209.s001.tiff]
